# Polarity and migration of cranial and cardiac neural crest cells: underlying molecular mechanisms and disease implications

**DOI:** 10.3389/fcell.2024.1457506

**Published:** 2025-01-06

**Authors:** Esteban Salinas, Francis Ruano-Rivadeneira, Juan Ignacio Leal, Teresa Caprile, Marcela Torrejón, Cecilia Arriagada

**Affiliations:** ^1^ Centro de Biología Celular y Biomedicina (CEBICEM), Facultad de Medicina y Ciencia, Universidad San Sebastián, Santiago, Chile; ^2^ Departamento de Ciencias Biológicas y Químicas, Facultad de Medicina y Ciencia, Universidad San Sebastián, Santiago, Chile; ^3^ Developmental Biology Laboratory 116, School of Biological Sciences, Faculty of Exact and Natural Sciences, Pontificia Universidad Católica del Ecuador, Quito, Ecuador; ^4^ Laboratory of Signaling and Development (LSD), Group for the Study of Developmental Processes (GDeP), Department of Biochemistry and Molecular Biology, Faculty of Biological Sciences, University of Concepción, Concepción, Chile; ^5^ Laboratory of Axonal Guidance, Group for the Study of Developmental Processes (GDeP), Department of Cellular Biology, Faculty of Biological Sciences, Universidad de Concepción, Concepción, Chile

**Keywords:** neural crest (NC), cell polarity, cell migration, neural crest disorder, cell signaling

## Abstract

The Neural Crest cells are multipotent progenitor cells formed at the neural plate border that differentiate and give rise to a wide range of cell types and organs. Directional migration of NC cells and their correct positioning at target sites are essential during embryonic development, and defects in these processes results in congenital diseases. The NC migration begins with the epithelial-mesenchymal transition and extracellular matrix remodeling. The main cellular mechanisms that sustain this migration include contact inhibition of locomotion, co-attraction, chemotaxis and mechanical cues from the surrounding environment, all regulated by proteins that orchestrate cell polarity and motility. In this review we highlight the molecular mechanisms involved in neural crest cell migration and polarity, focusing on the role of small GTPases, Heterotrimeric G proteins and planar cell polarity complex. Here, we also discuss different congenital diseases caused by altered NC cell migration.

## Introduction

Neural crest (NC) cells are multipotent progenitor cells vertebrate-specific (York and McCauley, 2020). They are induced during neurulation, at the neural plate border. After induction, NC cells differentiate into various cell types and tissues essential for vertebrate development (Mayor and Theveneau, 2013).

NC cells originate from the ectoderm but can also differentiate into mesodermal cell types. Traditionally, cell differentiation has been viewed as a gradual process of lineage restriction, where the potential of cells diminishes as the embryo develops. In this classical view, NC induction was considered a late embryonic event, closely tied to the formation of the neural tube. However, in chicken was found that NC cell induction could begin during or before gastrula stage (Basch et al., 2006). Interestingly, studies in *Xenopus* have revealed that key NC regulatory factors are expressed as early as the blastula stage in the animal pole, promoting pluripotency, suggesting that NC specification begins much earlier in development (Buitrago-Delgado et al., 2015). Additionally, a transient precursor population that expresses both canonical pluripotency transcription factors and neuroepithelial markers has been identified as giving rise to NC cells, supporting the notion that these cells possess pluripotent characteristics (Zalc et al., 2021). However, NC precursor cells exhibit a more restricted differentiation potential compared to embryonic stem cells (Prasad et al., 2020). Collectively, these findings suggest that NC specification is not a late, isolated event but rather a process that begins at the earliest stages of development and continues to be refined as the embryo undergoes further organization and forms structures such as the neural tube (Stuhlmiller and García-Castro, 2012; Schille and Schambony, 2017; Pla and Monsoro-Burq, 2018).

Major signaling factors, including the Bone Morphogenetic Protein (BMP), Wingless-related Integration Site (WNT), Fibroblast Growth Factor (FGF), Retinoic Acid (RA) and NOTCH, induce the formation of NC cells by establishing the neural plate and mesoderm. Initially, these cells are located at the edge of the neural plate, and during neurulation, the borders of the neural plate, known as neural folds, converge to form the neural tube. Subsequently NC cells migrate through the process of epithelial-mesenchymal transition (EMT) (Box1) to different parts of the embryo (Sauka-Spengler and Bronner-Fraser, 2008; Scholl and Kirby, 2009; Shih et al., 2017).

BOX 1 **Principal concepts of cell polarity and migration in neural crest cells.**

**Cell polarity**

Cell polarity refers to the asymmetric organization of cellular components, enabling cells to perform directed functions such as migration. This polarity is established and maintained through a network of interconnected positive feedback loops involving Rho family GTPases, phosphoinositide 3-kinases (PI3Ks), integrins, microtubules, and vesicular transport. Central to the regulation of cell polarity are the GTPase Rac and Cdc42, which are active at the front of migrating cells. These GTPases guide the formation of lamellipodia by positioning the microtubule-organizing center (MTOC) and Golgi apparatus toward the leading edge. The precise positioning of these structures facilitates polarized migration by ensuring that necessary vesicles and proteins are delivered to the leading edge, promoting forward protrusion and efficient cell movement (Ridley et al., 2003).

**Contact inhibition of locomotion (CIL)**

CIL is a process in which cells, upon encountering one another, form a transient contact, stop their movement, and then separate, redirecting their migration away from the point of contact. This mechanism is essential during embryonic development and cancer metastasis. In neural crest cells, CIL is established during the epithelial-to-mesenchymal transition (EMT), marked by a switch from E-cadherin to N-cadherin. This switch supports the reorganization of cellular protrusions and the redistribution of forces, allowing the cells to separate after contact. The small GTPase family plays a vital role in regulating this contact and separation process, ultimately ensuring effective directional migration via CIL (Scarpa and Mayor, 2016).

**Epithelial mesenchymal transition (EMT)**

EMT is a cellular process in which epithelial cells lose their defining characteristics and acquire mesenchymal features. This transition is crucial in various physiological and pathological contexts, including embryonic development, wound healing, and cancer progression. During EMT, cells undergo significant changes in their morphology, gene expression, and behavior. This process involves the downregulation of epithelial markers such as E-cadherin and the upregulation of mesenchymal markers such as N-cadherin. EMT is not a binary process but rather a spectrum of intermediate states, often referred to as partial, incomplete, or hybrid EMT states, where cells exhibit both epithelial and mesenchymal traits. These intermediate states are associated with increased cellular plasticity, invasiveness, and resistance to apoptosis, facilitating processes like tumor metastasis and therapy resistance (Pastushenko and Blanpain, 2019).

**Collective cell migration**

Collective cell migration is a fundamental biological process in which groups of cells move together in a coordinated manner. This mode of migration is crucial during embryonic development, tissue repair, and cancer metastasis. In this process, cells maintain stable or transient cell-cell adhesions, allowing them to move as cohesive units. Epithelial cells typically exhibit leader cells that form protrusions to guide follower, which maintain tight junctions. In contrast, mesenchymal cells form transient adhesions that direct their collective movement. The interaction with the extracellular matrix and the response to environmental cues are vital for the directional migration of these cell groups. Studies in various models, such as border cell migration in *Drosophila*, tracheal branching, and neural crest cell migration, highlight the conserved mechanisms of cell polarity, mechanical coupling, and chemotactic guidance that drive efficient collective movement (Scarpa and Mayor, 2016).

**Matrix stiffness**

Matrix stiffness is a critical factor influencing general cell migration and behavior. It refers to the rigidity of the extracellular matrix (ECM), which can vary from soft, as healthy tissues, to stiff, as in fibrotic or pathological conditions. Increased matrix stiffness affects cellular processes by altering the mechanical signals perceived by cells. Cells sense stiffness through mechanotransduction pathways involving integrins and the actin cytoskeleton, leading to changes in cell morphology, adhesion, and motility. Higher stiffness typically enhances cell migration by promoting focal adhesion formation and cytoskeletal reorganization, which are essential for generating the forces required for movement. Understanding how cells respond to varying stiffness in their microenvironment is crucial for elucidating the mechanisms underlying wound healing, tissue development, and various diseases, highlighting the role of mechanical cues in regulating cellular functions (Lopez-Cavestany et al., 2023).

**Placodes:** In the context of a review on Neural Crest Cells, “placodes” can be defined as transient columnar epithelia with neurogenic potential that develop in the ectoderm of the vertebrate head adjacent to the neural tube. These structures are crucial for the formation of paired sensory organs and cranial sensory ganglia, contributing to a wide variety of cell types, ranging from lens fibers to sensory receptor cells and neurons. Placodes originate from a common pre-placodal region, which is subsequently subdivided to generate specific types of placodes, following induction mechanisms that appear to be shared across all placodes (Graham and Begbie, 2000).

NC cells develop along the anteroposterior axis of the embryo and are categorized into four subpopulations: cranial, vagal, trunk, and sacral (Rothstein et al., 2018). While the dorsal neural tube typically serves as the main source of NC cells, the specific anteroposterior location along the neural tube from which NC cells arise varies by subpopulation. Cranial NC cells originate from the dorsal regions of the anterior neural tube (midbrain and hindbrain), vagal NC cells come from the caudal hindbrain), and trunk and sacral NC cells emerge more posteriorly, retaining a dorsal origin but with distinct regional characteristics. These distinctions highlight the significance of anteroposterior positioning in the specification and migration pathways of NC cells subpopulations. After migration, the NC cells differentiate into a wide variety of cells giving rise to tissues and organs, including the skeleton, glia, and melanocytes, among others (Martik and Bronner, 2017).

The specificity of NC cell migration was first identified using a quail-chick marker system. By creating chimeras between these two bird species, researchers were able to track the migration of a specific NC subpopulation and determine the tissues they eventually form (Le Douarin, 1973).

The cranial NC, which emerges at the border of the neural plate anterior to the 1^st^ somite goes through a remarkable transformation journey yielding diverse cell types such as the skeletal system and the peripheral cranial nerves, ocular structures, smooth muscles, and connective tissues of blood vessels. Additionally, cranial NC cells contribute to the dermis of the head, most of the melanocytes (excluding iris cells), and the meninges of the forebrain (Johnston et al., 1979; Le Douarin and Kalcheim, 1999; Creuzet et al., 2005; Noden and Trainor, 2005; Dupin et al., 2006; Mayor and Theveneau, 2013; Duband et al., 2015).

Vagal NC cells are located among somite 1st to 7th, between the cranial and trunk segments of the NC (Le Douarin and Teillet, 1974) and give rise to the neurons and supportive cells of the enteric nervous system along the entire digestive tract. They also form cardiac and dorsal root ganglia, as well as ectomesenchyme derivatives such as cartilage, connective tissue and bones (Nagy and Goldstein, 2017; Ganz, 2018).

Cardiac NC is a subdivision of the vagal NC and are responsible for the morphogenesis of the outflow region of the developing heart and the smooth muscle lining of blood vessels, contributing to the outflow valves (Schussler et al., 2021).

Trunk NC cells span from the 8th somite to the 28th somite and differentiate into neurons, glial cells of the peripheral nervous system, adrenal medulla, and the neurons and glial cells of the enteric nervous system (Green et al., 2017). Finally, the sacral NC extends from the 28th somite to the end of the embryo (Le Douarin and Kalcheim, 1999) and contributes to the enteric nervous system, forming the ganglia that innervate the hindgut. The sacral NC also migrates ventrally and colonizes the gut after the vagal NC (Wiese et al., 2017).

Each NC subpopulation migrates to specific destinations, where they contribute to forming a wide range of tissues and organs. This migration process is highly regulated and relies on maintaining proper cell polarity (Box 1), which is essential for their correct integration and function. Early investigations of NC cells were predominantly conducted on accessible amphibian and avian embryos, with mouse genetics later providing complementary insights. More recently, the zebrafish model has emerged as a valuable tool, offering unique advantages for studying NC cell dynamics (Rocha et al., 2020).

In this review, we focus on two essential subpopulations of the NC: the cranial NC and the cardiac NC. These branches have been instrumental in advancing our understanding of several diseases, such as cancer metastasis, craniofacial anomalies, and congenital heart defects. Research on cranial NC has shed light on the mechanisms driving craniofacial development and its associated disorders, while studies on cardiac NC have enhanced our knowledge of heart development and related anomalies.

### Cranial neural crest cell migration

The migration of cranial NC cells occurs after cells undergo to EMT. In *Xenopus* cranial NC cells, which are often used as a model for studying EMT in cancer, EMT takes place after induction at the neurula stage. As we mention before, during this transition, NC cells shift from an epithelial phenotype to a migratory mesenchymal state (Kelleher et al., 2006; Theveneau and Mayor, 2012b; Barriga et al., 2013). This process involves the loss of epithelial polarity, marked by a switch from E-cadherin to N-cadherin, which facilitates the cells migration into surrounding tissues (Kuriyama and Mayor, 2008; Sauka-Spengler and Bronner-Fraser, 2008; Steventon and Mayor, 2012; Theveneau and Mayor, 2012b). Following EMT, cranial NC cells migrate collectively via three distinct streams regulated by ephrin signaling, creating spatially defined paths called mandibular, hyoid and brachial (Kuriyama and Mayor, 2008; Gammill and Roffers-Agarwal, 2010; Theveneau and Mayor, 2012b; Theveneau and Mayor, 2012a; Nieto, 2013).

During migration, cell-cell interactions guide collective movement via mechanisms like contact inhibition of locomotion (CIL) and co-attraction (CoA), both crucial for directional migration (Carmona-Fontaine et al., 2008) (Box1). CIL reorients cells upon collision via Rho GTPase signaling and non-canonical Wnt pathways, which are crucial for maintaining migratory coherence (Carmona-Fontaine et al., 2008). Conversely, CoA mediated by C3a peptide, promotes cohesion counteracting dispersion (Carmona-Fontaine et al., 2011). NC cells secrete the complement factor C3a and express its receptor, C3aR (Carmona-Fontaine et al., 2011), resulting in high C3a concentrations in areas with dense NC cell populations, enabling cells that have lost contact with the group to migrate back along the chemotactic gradient. This process, known as CoA, involves C3a signaling leading to Rac1 activation, which polarizes the cells back toward the group (Carmona-Fontaine et al., 2011). The balance between CIL and CoA is critical for maintaining the collective nature of migration (Theveneau et al., 2010; Woods et al., 2014). Inhibiting C3 or its receptor reduces cell collectiveness, as CIL drives the cells apart, hindering their ability to migrate efficiently towards a chemoattractant source (Carmona-Fontaine et al., 2011; Woods et al., 2014).

**FIGURE 1:**
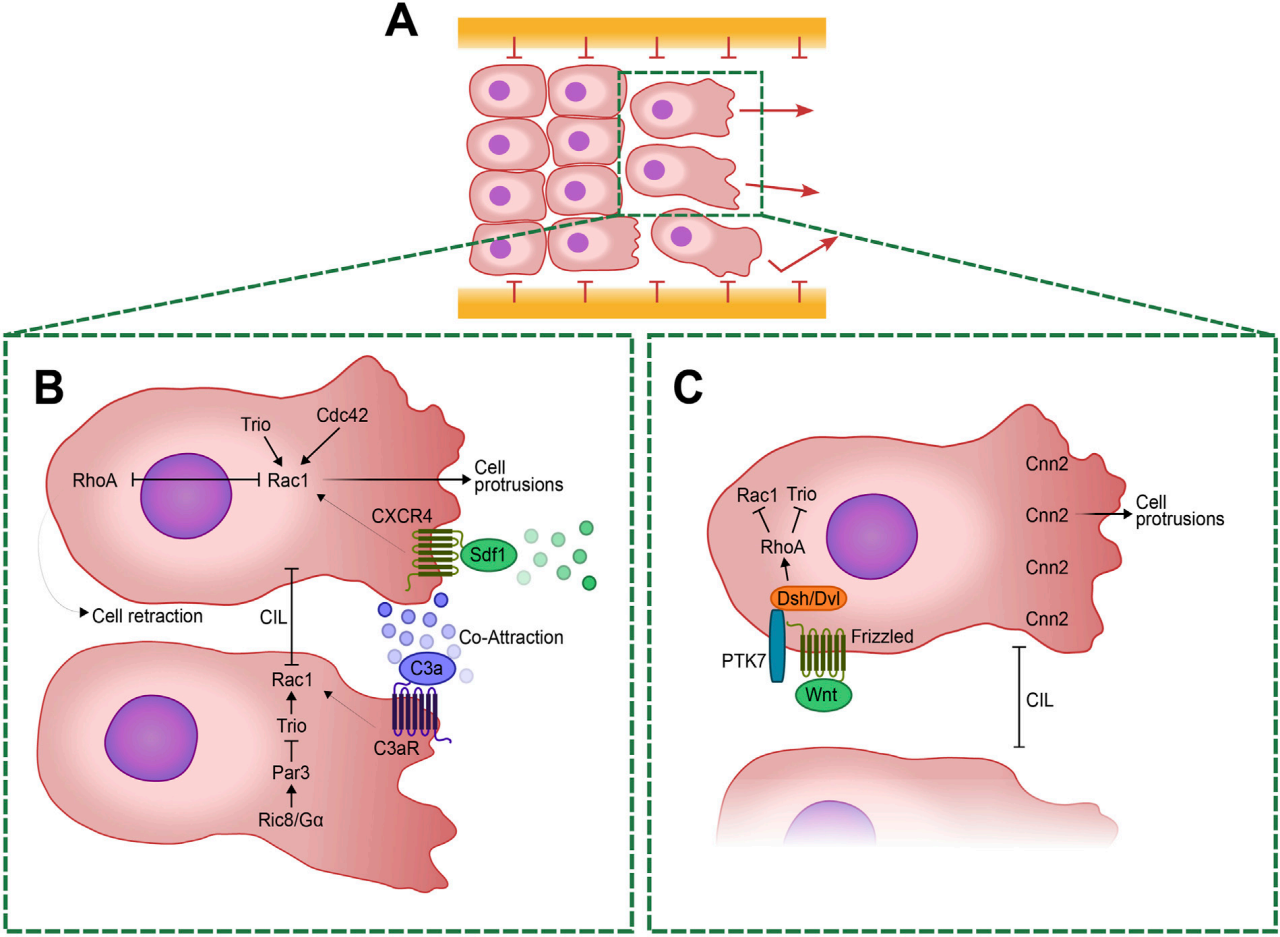
Cellular and Molecular mechanisms regulating Neural Crest (NC) cell migration and polarity. **(A)** Schematic representation of a group of collectively migrating NC cells with polarized leader cells at the forefront. The *in vivo* migration routes are delineated by confinement boundaries expressing different members of the ephrin/Eph family. A highlighted box indicates two of the leader cells in the group, expanded in **(B, C)**. **(B)** Enlarged view of the leader cells showing the antagonistic regulation of RhoA and Rac1. RhoA facilitates cellular retraction at the posterior edge, while Rac1 promotes protrusions at the anterior edge. Rac1 activity is modulated by several pathways including Trio and Cdc42. The CXCR4/Sdf1 signaling pathway, originating from placodal cells, guides the migratory trajectories of NC cells. Meanwhile, C3a signaling, released by the NC cells themselves, helps maintain their cohesion through a mechanism of co-attraction by regulating Rac1 activity. Contact Inhibition of Locomotion (CIL) is driven by Rac1 inhibition at the cell-cell contacts. This process is intricately regulated by the activation of Ric8A, which activates Gα proteins. Then Gα proteins activates Par3 that inhibits Trio at cell-cell contacts, leading to a specific localized Rac1 inhibition and ensuring proper cell retraction. **(C)** Planar Cell Polarity (PCP) at the leading edge is regulated by the WNT signaling pathway, which activates Frizzled receptors. This, in turn, activate Dishevelled (Dsh/Dvl) and PTK7, leading to the downstream activation of RhoA. RhoA inhibits Trio and Rac1 to ensure proper cell polarity. Calponin-2 (Cnn2) at the leading edge induces protrusion formation necessary for cell migration. This regulation is crucial for maintaining the balance between cell protrusion and retraction, enabling directed migration.

Stromal cell-derived factor 1/CXC Chemokine Receptor 4 (Sdf1/CXCR4) signaling further directs migration by enabling gradient sensing, stabilizing protrusions and activating Rac-1 at leading edges (Belmadani et al., 2005; Olesnicky Killian et al., 2009; Theveneau et al., 2010) (Figure 1). Sdf1 is produced by placode cells (Box 1), an epithelial tissue crucial for the formation of sensory organs. This tissue employs a “chase and run” mechanism, wherein cranial NC cells chase placode cells secreting Sdf1 via chemotaxis. Upon contact, placode cells retreat, eliciting a heterotypical CIL response in NC cells (Theveneau et al., 2013; Szabó and Mayor, 2015). Additionally, ephrins restrict cell entry into specific regions, ensuring stream integrity (Smith et al., 1997; Helbling et al., 1998).

Cranial NC collective migration is regulated by both, the molecular signals as we described above and mechanical cues (Box 1) in their environment (Carmona-Fontaine et al., 2008; Theveneau et al., 2010; Barriga et al., 2018). Together, these intricate mechanisms, encompassing both mechanical and molecular signals, coordinate the migration of cranial NC cells, underscoring the complex interplay between these cues and cellular responses crucial for embryonic development.

### Cardiac neural crest cell migration

Cardiac NC cell developmental processes overlap with the segmentation of rhombomeres (R1-R8) (Odelin et al., 2018). Due to differences in migration patterns, cardiac NC cells from R1-R4 take the name of pre-otic cardiac NC cells and from R6-R8 are called post-otic cardiac NC cells (Taneyhill and Schiffmacher, 2017; Piacentino et al., 2020).

Post-otic cardiac NC cells will migrate towards the pharyngeal arches 3, 4 and 6. From these arches, a subset of cells continues their migration into the heart, where they contribute to outflow tract (OFT) septation or form the parasympathetic ganglia of the heart. The cardiac NC cells migrate from the hindbrain in three “streams”: cranial (first), medial (second) and caudal (third) (Trainor et al., 2002). For a correct migration and differentiation, cardiac NC cells secrete proteases, including matrix metalloproteases (MMP) (Cai and Brauer, 2002; Komatsu et al., 2007; Arai et al., 2019). Studies have demonstrated the essential role of certain proteases in regulating cardiac NC cells migration and differentiation (Komatsu et al., 2007; Wagner et al., 2010; Chen et al., 2015; Arai et al., 2019). For instance, it has been found that the MMP inhibitor, KB8301, decrease the migration of cardiac NC cells (Wagner et al., 2010). Additionally, research on mice found that depletion of ADAM19 protease results in defects in the ventricular septum and heart valves (Komatsu et al., 2007). Additionally, using Wnt1-Cre lineage to specifically deplete ADAM19 in NC cells, it was found that ADAM19 is required for the proper cardiac NC cell fate and to avoid abnormal cardiac chondrogenesis (Arai et al., 2019).

Several mechanisms govern the correct migration of cardiac NC cells into their respective pharyngeal arches. One of these mechanisms involves the T-box transcription factor Tbx1, which regulates the Slit ligand and its receptor roundabout (ROBO) expressed on cardiac NCs, allowing its migration and cardiac OFT elongation (Calmont et al., 2009). Additionally, endothelin-A receptors (Fritz et al., 2019), members of the TGFβ superfamily (Scholl and Kirby, 2009) and subtypes α and β of platelet-derived growth factor (PDGF) are implicated in this process (Scholl and Kirby, 2009; Dinsmore and Soriano, 2022).

Furthermore, FGF8 drives the migration of cardiac NC cells from the third migration stream. Numerous studies have underscored the pivotal role of FGF8 in ensuring the survival and proper migration of cardiac NC cells, specifically to pharyngeal arches 3, 4, and 6 (Sato et al., 2011). FGF8 is also crucial for the development of structures derived from the embryonic mesoderm and endoderm (Itoh et al., 2016). This growth factor not only facilitates the migration but also supports the differentiation and integration of cardiac NC cells into the developing cardiovascular system, highlighting its essential function in embryonic development.

It was found that three streams of cardiac NC cells migrate separated by two regions, rhombomere 3 and 5. Apoptosis of premigratory NC cells of rhombomere 3 and 5 is important for defining the separation and migration of NC cells, as they avoid mesenchymal irruption of the mentioned rhombomeres (Graham et al., 1993; Graham et al., 1993; Adams et al., 1996; Kulesa and Fraser, 1998; 2000; Ellies et al., 2002). BMP4 and MSX-2 appear to be involved in this process by inducing apoptosis (Graham et al., 1993; Graham et al., 1994).

The directionality of cardiac NC cells migration, similar to cranial NC cells, is partially regulated by guidance cues from certain protein families, such as semaphorins and ephrins, which have the ability to attract or repel cells depending on the membrane receptor. The NC cells express the semaphorin receptors neuropilin-1 and 2 and the coreceptor Plexin- D1 and Plexin-A2. This set of receptors allows the NC cells to react to different types of semaphorins and is crucial in the separation of the aortic root and pulmonary trunk during truncus arteriosus mediated by NC cells (Brown et al., 2001; Gitler et al., 2004; Toyofuku et al., 2008; Kodo et al., 2017; Yamagishi, 2021).

In this way, Semaphorins 3A, 3F and 6, are expressed in the lateral pharyngeal mesenchyme and in the dorsal neural tube and repel NC cells through their interaction with Plexin-A2 and neuropilin receptors (Eickholt et al., 1999; Osborne et al., 2005; Toyofuku et al., 2008; Yamagishi, 2021). This repulsion drives NC cells to the OFT that expresses Semaphorin 3C and attract NC cells through PlexinD1/Neuropilin1 receptors. As a result of the differential tissue expression of semaphorin members, the NC cells migrate along the dorsal region and pharyngeal arches until they reach the OFT, the final destination, where they differentiate into endocardial and smooth muscle cells (Zhang et al., 2021).

Semaphorin 3C is regulated by the transcription factors Foxc1/C2, which promote its expression in the OFT, and by Tbx1 and FGF8, which inhibit its ectopic expression in the pharyngeal arches (Kodo et al., 2017).

The mechanism used by Semaphorin 3A to repel NC cells depends on the inhibition of Rac1, RhoA, or Cdc42 activity, reducing cell protrusion and affecting cell migration (Bajanca et al., 2019). On the other hand, Piezo1, a mechanosensitive channel, is necessary for Rac1 inhibition. In the absence of Piezo1, Semaphorin 3A inhibition alone is insufficient to prevent NC cell migration (Canales Coutiño and Mayor, 2021).

In addition to the semaphorin family members, the guidance molecules belonging to the ephrin family play also a dual role in cell migration by binding to tyrosine kinase receptors, which in turn reduce the activity of cadherins responsible for cell adhesion (Cayuso et al., 2015). The ephrin receptors can either induce heterotypic tension or repulsion, while E-cadherins neutralize homotypic tension (Fagotto et al., 2014; Rohani et al., 2014; Canty et al., 2017). This intricate balance of tension dynamics influences the separation of the cardiac NC cells during development. By regulating the tension levels between different cell types and within cell populations, the ephrin family and their receptors facilitate the migration of cardiac NC cells towards target locations, such as the pharyngeal arches, ensuring proper embryonic development (Batlle and Wilkinson, 2012; Fagotto et al., 2014; Rohani et al., 2014; Cayuso et al., 2015; Canty et al., 2017; Fagotto, 2020).

Once the cardiac NC cells reach the pharyngeal arch they differentiate into smooth muscle and a subset of these cells cluster into the OFT, which undergo ECM remodeling to form the base of the aorta and pulmonary artery (Eisenberg and Markwald, 1995; Plein et al., 2015). Moreover, cardiac NC cells give rise to the formation of endocardial cushions that coalesce to form the pulmonary aortic septum (Bergwerff et al., 1998; Waldo et al., 1998).

Cell polarity stands out as a fundamental determinant of cell migration, enabling cells to adeptly sense environmental cues, interpret signals, and execute directed movement crucial for developmental processes, tissue regeneration, and other vital biological functions (Rauzi et al., 2008; Weber et al., 2012; Roca-Cusachs et al., 2013; Shindo and Wallingford, 2014).

In the subsequent section of this review, we will delve into the intricate molecular mechanisms that orchestrate cell polarity, particularly focusing on its role during the migration of NC cells.

### Cell polarity during neural crest cell migration

The establishment and maintenance of cell polarity is essential to ensure the directional movement and effective response to environmental cues. During cranial NC cell migration, cell polarity plays a crucial role in orchestrating the dynamic process of EMT and subsequent migration into adjacent tissue. Key mechanisms governing collective cranial NC cell migration, as we mention above, include CIL, CoA, and chemotaxis, all of which are regulated by localized activity of small GTPases.

Actin filaments and microtubules serve as central regulators of cell shape and motility, crucial for the formation of cell protrusions at the leading edge and adhesion to the extracellular matrix (ECM) during migration (Le Clainche and Carlier, 2008; Etienne-Manneville, 2014). The coordination between these cytoskeletal elements is mediated by small GTPases of the Rho family (Rac1, Cdc42, and RhoA), which govern processes like cell polarity, actin polymerization, and actomyosin contractility through spatiotemporal activation mechanisms (Rodriguez et al., 2003; Lawson and Ridley, 2018). During *Xenopus* cranial NC cells migration *ex vivo*, these GTPases exhibit distinct spatial activities: Rac1 is highly active at the front, while RhoA predominates at the rear, crucial for directional movement (Carmona-Fontaine et al., 2008; Matthews et al., 2008; Clay and Halloran, 2010; Theveneau et al., 2010; Leal et al., 2018).

Activation of Rac1 is crucial for initiating lamellipodia and membrane ruffles by triggering downstream proteins like WAVE and Arp2/3, which facilitate actin polymerization at the cell leading edge (Parri and Chiarugi, 2010). Conversely, Rho signaling pathway activates ROCK (Rho-associated serine/threonine kinase), which in turn phosphorylates the myosin regulatory light chain, thereby regulating stress fiber formation and controlling cell contraction and focal adhesion assembly (Parri and Chiarugi, 2010; Spiering and Hodgson, 2011). Notably, these GTPases—Rac1, Cdc42, and RhoA—reciprocally regulate each other activity; for instance, Cdc42 activation stimulates Rac1 while inhibiting RhoA, and *vice versa*, thereby coordinating cytoskeletal dynamics and cellular responses (Spiering and Hodgson, 2011). This spatial regulation of small GTPases provides a robust readout for studying cell polarity during migration. Moreover, Par3 in *Xenopus* negatively modulates Rac1 at cell-cell contacts by inhibiting the Rac-GEF Trio, thereby influencing microtubule dynamics and contributing to CIL during cranial NC migration (Moore et al., 2013) (Figure 1).

This polarization of GTPase activity is regulated by CIL, where cell-cell contact inhibits protrusions in trailing cells, ensuring directional migration of NC cells (Mayor and Carmona-Fontaine, 2010; Stramer et al., 2013). This process involves RhoA activation and Rac inhibition at cell contacts, promoting retraction and polarization necessary for collective migration (Carmona-Fontaine et al., 2008; Matthews et al., 2008; Theveneau et al., 2010). Despite the tendency for cell dispersion under contact inhibition conditions, NC cells are held together by negative signals and chemoattractants, facilitating their cohesive directional migration (Carmona-Fontaine et al., 2011).

Additionally, it was demonstrated that cranial NC cells in chicken embryos migrate by progressively refining the polarity of their protrusions. This strategy sacrifices CIL to efficiently explore their environment and maintain precise migration despite the noisy guidance signals present around them. This mechanism of searching and selecting productive protrusions contrasts with previous models, such as those observed in *Xenopus*, where cells within the migratory stream lack polarity and protrusions (Genuth et al., 2018).

On the other hand, during migration it was demonstrated that chicken cranial NC cells release extracellular vesicles, including exosomes and migrasomes, which are critical for their migration. Inhibiting exosome release resulted in less polarized and more rounded cells, leading to loss of directional migration and reduced speed. These findings highlight the importance of vesicle-mediated communication in collective cell polarity and migration, offering key insights into polarity mechanisms in cranial NC cells (Gustafson et al., 2022).

An additional mechanism that involves cell polarity was found in mice knockout (KO) for Connexin 43 (Cx43), a gap junction protein. NC cells and epicardial cells express Cx43, and Cx43 KO mice shown an abnormal coronary artery patterning and outflow obstruction, suggesting a problem in NC cells migration (Lo et al., 1997; Li et al., 2002; Walker et al., 2005; Clauss et al., 2006). Posterior studies using embryonic fibroblasts from Cx43 KO mice found that Cx43 deficiency leads to cell polarity defects characterized by the failure of the Golgi apparatus and microtubules orientation in the direction of wound closure (Francis et al., 2011).

### Heterotrimeric G protein controlling cell polarity during neural crest cell migration

Recent studies highlight the role of heterotrimeric G proteins in controlling cell migration across various developmental contexts. Members of the Gα subunit family, including Gα12/13, Gαi/o, Gαq/11, and Gαs, are implicated in orchestrating signaling cascades that promote actin cytoskeleton reorganization via regulation of small GTPases (Nobes and Hall, 1995; Kjøller and Hall, 1999; Sah et al., 2000; Rohde and Heisenberg, 2007; Cotton and Claing, 2009). These G proteins initiate signaling upon ligand binding to their receptors, which act as guanine nucleotide exchange factors (GEFs), catalyzing the exchange of GDP for GTP on Gα subunits to activate downstream effectors (Gilman, 1987). Notably, Ric-8 acts as a GEF-independent regulator that accelerates this nucleotide exchange, thereby maintaining Gα subunits in an active signaling state (Klattenhoff et al., 2003; Siderovski and Willard, 2005; Hinrichs et al., 2012).

In the context of polarity, research in *Caenorhabditis elegans* embryos and *Drosophila* neuroblasts has demonstrated that Gαi and Gαo subunits from the heterotrimeric G protein, are key regulators of apicobasal polarity, functioning independently of receptors (Bellaiche and Gotta, 2005; Siderovski and Willard, 2005). This polarity is crucial for processes such as asymmetric cell division and tissue formation, and its disruption is implicated in diseases like cancer (Hirose et al., 2006; Feigin and Muthuswamy, 2009; Knoblich, 2010). During asymmetric cell division, Gαi engages in a non-canonical pathway that controls mitotic spindle orientation by influencing microtubule behavior. Studies involving *C. elegans* embryos, *Drosophila* neuroblasts, and sensory organ precursors have shown that proper spindle orientation is essential for generating daughter cells with different sizes and functions (di Pietro et al., 2016). Specifically, in *C. elegans*, a complex involving the Gαi subunit contributes to spindle positioning by creating an imbalance in cortical forces (Grill et al., 2001). This conserved complex, comprising Gαi, LGN, dynein/dynactin, and NuMA, is crucial for correctly aligning the spindle (Kiyomitsu, 2019; Poon et al., 2019).

In *Drosophila* neuroblasts, the Par3/αPKC/Par-6 complex, along with Gαi and Pins, establishes apicobasal polarity during the initial asymmetric division (di Pietro et al., 2016). In mammals, the interaction between Gαi, LGN, and NuMA with astral microtubules is essential for proper spindle alignment (Du and Macara, 2004; Woodard et al., 2010). Additionally, Gβγ has a role in spindle asymmetry in *Drosophila*, though its interaction with Gαi/Pins requires further elucidation (Fuse et al., 2003). Gαi isoforms and their regulatory partners have also been identified at centrosomes, influencing various polarity processes, including apicobasal polarity through interaction with proteins like vesicle-associated protein (GIV), a GEF for Gαi (Sasaki et al., 2015). This coordinated regulation of Gαi signaling underscores its importance in asymmetric cell division, crucial for development, tissue regeneration, and disease progression, such as in cancer. The chaperone Ric-8, a conserved cytosolic GEF initially identified in *C. elegans* and *Drosophila* is necessary for forming the Gα/GPR-1/2 complex and ensuring correct localization of Gα and related proteins, which are critical for asymmetric spindle orientation during asymmetric cell division (Miller and Rand, 2000; Afshar et al., 2004; Afshar et al., 2005; Couwenbergs et al., 2004; David et al., 2005; Hampoelz et al., 2005).

In *Drosophila* neuroblasts and sensory organ precursor cells, Ric-8 is essential for accurate spindle orientation, asymmetric localization of cell-fate determinants, and regulating daughter cell size. Ric-8 also plays a key role in gastrulation, a process that depends on receptor-mediated G-protein signaling. Notably, in the absence of Ric-8, G-protein subunits, including Gαi, Gαo, Gβ, and likely Gγ, are unable to localize to the cell cortex. This finding suggests that Ric-8 may function not only as a GEF but also as a facilitator of Gα activity by promoting the assembly of heterotrimeric G-proteins (Matsuzaki, 2005).

In *Xenopus*, Ric-8A is prominently expressed during cranial NC formation and migration, including derivative tissues like craniofacial arches (Maldonado-Agurto et al., 2011). Additionally, Ric-8A is essential for orchestrating proper migration by modulating cell adhesion and chemotaxis through its dual functions as a GEF and chaperone for the subunit Gα13 (Fuentealba et al., 2013; Toro-Tapia et al., 2018). As we mention above, studies have demonstrated that Ric-8A interacts with Gα subunits to localize proteins like LGN, Numa, and dynein at the cell cortex, crucial for spindle positioning during division in mammals (Woodard et al., 2010). An investigation utilizing GTPase-based probes in live cranial NC cells demonstrate that Ric-8A levels critically regulate the localization of active Rac1 and RhoA during migration (Leal et al., 2018).

In migrating cranial NC cells, Ric-8A depletion leads to aberrant localization of aPKC and Par3, disrupting Rac1 inhibition at cell-cell contacts and impairing cell response to CIL (Moore et al., 2013; Leal et al., 2018). Furthermore, as we mention above Ric-8A modulates Rac1 and RhoA active localization, affecting cytoskeletal dynamics and cell morphology essential for directional migration (Leal et al., 2018; Toro-Tapia et al., 2018; Toro-Tapia et al., 2017). These findings propose that Ric-8A governs cranial NC cell polarity via heterotrimeric G-protein signaling pathways, highlighting its critical role in regulating cell adhesion and chemotaxis during migration (Fuentealba et al., 2013; Toro-Tapia et al., 2018; Toro-Tapia et al., 2017). Future investigations should focus on identifying specific Gα subunits and GPCRs involved in this intricate signaling cascade.

### The loss of apico-basal polarity is essential for neural crest migration

After their induction and specification, NC cells initially exhibit an epithelial phenotype, characterized by strong apico-basal polarity, where they are tightly connected through cell junctions like E-cadherin and occludin (Kandel et al., 2000). This polarity is a key feature of epithelial cells, which are stable, organized in sheets, and supported by a basal lamina.

The transition of NC cells from an epithelial to a mesenchymal state, known as EMT, involves a loss of apico-basal polarity and the acquisition of front-rear polarity. This change is marked by the downregulation of epithelial junction proteins and the upregulation of mesenchymal markers, leading to reduced cell-cell adhesions and increased motility (Hay, 1995; Vandewalle et al., 2005).

BMP signaling, along with transcription factors such as Snail, Slug and Twist, play crucial roles in this process by repressing epithelial markers like E-cadherin, thereby disrupting cell junctions and promoting the mesenchymal phenotype (Kang and Massagué, 2004; Taneyhill et al., 2007; Rogers et al., 2013). This reorganization allows the NC cells to detach from the neural tube and become migratory (Theveneau et al., 2010). In addition to its roles in induction and delamination, BMP signaling activates the transcriptional repressor Sip1 in NC cells, promoting EMT (Kang and Massagué, 2004; van Grunsven et al., 2007; Thiery et al., 2009; Kerosuo and Bronner-Fraser, 2012; Rogers et al., 2013). Sip1 targets genes that regulate epithelial cell-cell junctions, notably suppressing E-cadherin expression, which is crucial for normal NC cell migration (Vandewalle et al., 2005; van Grunsven et al., 2007; Rogers et al., 2013). Delamination may also require a brief inhibition of WNT signaling (Rabadán et al., 2016).

A critical aspect of EMT in the NC is the “cadherin switch,” where the cells transition from expressing E-cadherin to N-cadherin, which is essential for their migration (Dady et al., 2012; Rogers et al., 2013). In NC cells during migration, cadherins, such as cadherin-7 and cadherin-11, are also expressed (Hadeball et al., 1998; Nakagawa et al., 2001; Cheung et al., 2005), while residual levels of E-cadherin persist (Barriga et al., 2013). Contrariwise, in trunk NC cells, the “cadherin switch” continues as they transition to cadherin-6 and cadherin-7, further promoting migration (Nakagawa and Takeichi, 1995; Park and Gumbiner, 2012). Throughout EMT, NC cells undergo significant changes in their cellular architecture, driven by alterations in cytoskeletal organization and cell junction dynamics. These changes are essential for the cells transition from a stationary, epithelial state to a mobile, mesenchymal one, facilitating their migration during development (Thiery et al., 2009; Nieto and Cano, 2012).

After discussing how heterotrimeric G proteins control cell polarity during NC migration, it becomes crucial to address another foundational aspect of NC migration: the loss of apico-basal polarity. This loss is essential for enabling the EMT that transforms NC cells from a stationary, polarized state to a migratory, mesenchymal phenotype. However, while the breakdown of apico-basal polarity allows for individual cell motility, effective NC migration also depends on coordinated collective movement (Nieto and Cano, 2012). This brings us to the role of Planar Cell Polarity (PCP) signaling, which regulates the interactions between cells within the migrating NC population. By modulating these cell-cell interactions, PCP signaling ensures that the directional migration of NC cells remains organized and coherent (Theveneau et al., 2010), highlighting the complex interplay between different polarity mechanisms during NC migration.

### Planar cell polarity during neural crest migration

Although PCP signaling has been primarily studied in other cellular contexts, evidence suggests that cell polarity in cranial NC cells is also regulated by WNT/PCP signaling pathway, which is essential for the directed migration of these cells. This pathway regulates cell orientation within the tissue plane and coordinates their collective movement, crucial for their dispersion throughout the embryo (Theveneau et al., 2010).

Disruption of this pathway has been shown to inhibit the migration of both cranial and trunk NC cells, demonstrating its importance in the process (De Calisto et al., 2005; Carmona-Fontaine et al., 2008; Shnitsar and Borchers, 2008).

The WNT signaling pathway orchestrates a wide range of biological processes throughout development and adulthood (Clevers and Nusse, 2012). This pathway operates through two main branches: the canonical and non-canonical WNT pathways, both of which involve WNT ligands binding to receptor complexes on the cell membrane. In the canonical signaling pathway, the absence of WNT ligands, β-catenin is phosphorylated by GSK3β and targeted for degradation. Conversely, activation of the canonical pathway inhibits GSK3β, stabilizing β-catenin, allowing it to translocate into the nucleus where it interacts with LEF/TCF transcription factors to regulate gene expression, often leading to cell differentiation (Aberle et al., 1997; Angers and Moon, 2009). Recent studies have identified a novel mechanism within canonical WNT signaling, known as Wnt-STOP (Wnt-induced stabilization of proteins), which functions independently of β-catenin and does not require new protein synthesis (Albrecht et al., 2021). In this pathway, WNT signaling rapidly sequesters glycogen synthase kinase 3 (GSK3) into multivesicular bodies (MVBs) and lysosomes, preventing it from phosphorylating target proteins and thereby protecting them from ubiquitination and degradation. This process, mediated by the endosomal sorting complexes required for transport (ESCRT) machinery, stabilizes a significant portion of cellular proteins, including key regulators of cell growth and metabolism. Unlike traditional WNT signaling that relies on β-catenin, the Wnt-STOP mechanism promotes rapid cellular responses such as increased lysosomal activity, macropinocytosis, and anabolic metabolism, supporting cell proliferation and survival (Taelman et al., 2010; Dobrowolski and De Robertis, 2012; Vinyoles et al., 2014).

On the other hand, the non-canonical WNT pathways act also independently of β-catenin and include pathways regulating intracellular calcium levels and small G-proteins such as Rho/Rac, which control PCP through remodeling of the actin cytoskeleton. PCP signaling, characterized extensively in *Drosophila*, involves protein sets like Flamingo (known as Cadherin EGF LAG seven-pass-G type receptor Celsr or Fmi), Van Gogh-like (Vangl), Prickle (Pk), Frizzled (Fz), Dishevelled (Dsh/Dvl), Dishevelled-associated activator of morphogenesis (Daam), which establish subcellular asymmetry through interactions at cell boundaries. These mechanisms generate planar polarity crucial for diverse processes in vertebrates, including axis elongation, neural tube closure, and directional cell migration (Axelrod, 2009; Vladar et al., 2009; Bayly and Axelrod, 2011; Gray et al., 2011; Wallingford, 2012) (Figure 1).

PCP signaling, orchestrates cell orientation within epithelial tissues through asymmetric distribution of PCP proteins like Dsh/Dvl and Fz (Shindo and Wallingford, 2014). Initially studied in insect wing and cuticle development, PCP proteins influence diverse vertebrate structures such as mammalian hair follicles and vertebrate hair cells, where they govern the orientation of stereocilia and basal bodies (Lawson and Ridley, 2018). Beyond structural orientation, PCP signaling regulates critical cellular processes like convergent extension during gastrulation and neural tube closure by modulating actin cytoskeleton asymmetry through Rho GTPases like Rac and RhoA (Axelrod and McNeill, 2002; Vladar et al., 2009; Tissir and Goffinet, 2010; Wallingford, 2012).

NC cells migrate in organized streams where the leading cells exhibit the most directional persistence and active protrusions. PCP signaling ensures that only the leading edge of these cells is allowed to extend protrusions, while other cell surfaces are restrained, thereby coordinating group movement (Carmona-Fontaine et al., 2008; Theveneau et al., 2010). When this signaling is disrupted, NC cells lose their coordinated movement and instead produce protrusions randomly, resulting in inefficient migration (Carmona-Fontaine et al., 2008).

Recent studies demonstrate that PCP elements localize at cell contacts during NC migration, where they inhibit Rac and activate RhoA upon cell collision, crucial for cell repulsion (Carmona-Fontaine et al., 2008; Clay and Halloran, 2010; Theveneau et al., 2010) (Figure 1). Proteins like PTK7 and WNT11 facilitate Dsh/Dvl recruitment to cell membranes during this process, underscoring their role as essential regulators of PCP signaling in NC migration across diverse vertebrate species (Carmona-Fontaine et al., 2008; Shnitsar and Borchers, 2008; Theveneau et al., 2013). Interestingly, while these components exhibit specific localization patterns in NC cells, their distribution can vary in other cell types, suggesting that certain PCP mechanisms might be conserved across different cellular contexts (Luga et al., 2012; Kaucká et al., 2015; Zhang et al., 2016). However, less is known about the roles of other PCP elements, such as Vangl, Pk, and Celsr, in the context of NC cell migration, particularly in mammals, where NC migration appears less clearly dependent on PCP signaling (Sasselli et al., 2013; Pryor et al., 2014). While NC migration appears unaffected in both constitutive *Vangl2* mutants and conditional mutants where *Vangl2* is deleted throughout the NC lineage (Pryor et al., 2014), *Celsr3* and *Fzd3* are essential for proper gut innervation by NC-derived enteric neurons, indicating these genes have a more refined role in NC development (Sasselli et al., 2013). PCP mutations can lead to severe neural tube defects (NTDs) such as craniorachischisis (Murdoch et al., 2003), demonstrating its importance in neural tube morphogenesis. However, its role in the NC itself, especially in mammals, may not be as critical as in the neural tube, indicating that more research is needed to fully understand its functions across different neural crest populations.

Moreover, downstream effectors like calponin-2 (Cnn2) further link PCP-mediated RhoA and Rac regulation to actin dynamics, critical for proper NC migration and tissue formation. Cnn2 is involved in the dynamic organization of the actin cytoskeleton in migratory NC cells. Cnn2 is inhibited downstream of non-canonical WNT signaling and polarized in the leading edge. Cnn2 polarization in the leading edge leads the formation of directed protrusions in explants and is required for directed migration of NC cells *in vivo* (Ulmer et al., 2013) (Figure 1).

Additionally, Dsh/Dvl2 mutants exhibit neural tube and cardiovascular defects, including double outlet right ventricle (DORV), transposition of the great arteries (TGA), and persistent truncus arteriosus (PTA), associated with abnormalities during OFT septation. Since the NC cells marker Pitx2 was barely detected in the OFT of Dsh/Dvl2 mutants, it is suggested that the cardiovascular defects are due to altered cardiac NC cell migration (Hamblet et al., 2002).

Thus, PCP signaling conservation in regulating directional migration underscores its fundamental role in NC development across different vertebrate organisms (Carmona-Fontaine et al., 2008; Matthews et al., 2008; Banerjee et al., 2011; Rios et al., 2011; Theveneau et al., 2013).

It has been demonstrated that Prickle1a and Prickle1b proteins, components of the PCP signaling pathway, are essential for the proper polarization and migration of cranial NC cells in zebrafish. Mutations in either or both genes result in aberrant polarization along the antero-posterior axis of the embryo, instead of the normal lateral orientation, thereby affecting the direction and efficiency of cell migration. This shift in polarity axis alters the migration direction, which is intriguing as other PCP-deficient conditions simply lose polarity and motility. The Prickle1-deficient condition also exhibited abnormal levels of cadherins and prolonged blebbing. These findings underscore the importance of Prickle1 in regulating cell polarity, emphasizing that correct polarization is crucial for coordinated cell migration during embryonic development (Ahsan et al., 2019).

Interestingly, it was described that mutants with a Prickle1-missense allele, named *Beetlejuice* (*Bj*) shown defects in cell polarity and migration causing congenital heart defect, including short OFT phenotype, skeletal and craniofacial anomalies (Liu et al., 2014; Gibbs et al., 2016).

PCP proteins accumulate at cell contact regions, but whether they display asymmetric distribution between colliding cells is not fully understood. While asymmetry in PCP proteins is well-documented in various organisms, its role in NC cell migration requires further investigation. Key elements like Fz and PTK7 recruit Dsh/Dvl to these regions, yet the precise mechanisms linking localized Dsh/Dvl distribution to Rho activity regulation remain unclear (Carmona-Fontaine et al., 2008; Shnitsar and Borchers, 2008; Theveneau et al., 2013).

In addition, PCP signaling is influenced by external factors like diffusible signals (e.g., Sdf1/Cxcl12) and physical interactions between non-adjacent NC cells, by long range filopodia structures (Teddy and Kulesa, 2004; Theveneau et al., 2010). These findings suggest that the significance of PCP signaling in NC migration may differ between species, reflecting the complexity and variability of the mechanisms involved.

In summary, while PCP signaling is a key player in many developmental processes, its role in NC cells seems to be limited or context-dependent, with more robust evidence in lower vertebrates compared to mammals.

### Surrounding context stiffness affect cell polarity and migration

ECM can be classified into two types: 1. the basement membrane, which consists of a dense network of core proteins such as laminin, collagen IV, nidogen, perlecan (Hspg2), and agrin, and 2. the interstitial matrix, which is a varied combination of elastin (Eln), fibronectin (Fn1), collagens, and proteoglycans such as Aggrecan (Acan) and Versican (Vcan) (Frantz et al., 2010). The proportion of these proteins will determine the final stiffness of the ECM (Theocharis et al., 2016) (Box1). Specific ECM composition and remodeling are controlled by a group of proteins, including MMPs, adamalysins (ADAMs/ADAMTSs), and hyaluronidases, which regulate the ECM degradation (Bonnans et al., 2014).

The ECM diversity is created by spatiotemporal regulation of the ECM production, modification, and degradation processes (Bonnans et al., 2014). The interaction between cells and the ECM is crucial during embryogenesis (Hynes and Naba, 2012) and it plays a pivotal role during NC cell migration (Perris and Perissinotto, 2000). For instance, genes encoding proteases that regulate ECM homeostasis and ECM proteins are expressed in migrating cells of both *Drosophila* and chicken (Bae et al., 2017). This suggests that it is a highly conserved mechanism facilitating cell migration, which can be extrapolated to the context of NC cell migration in vertebrates (York and McCauley, 2020).

Initially, to facilitate the EMT, NC cells degrade the basal extracellular matrix (ECM) at the dorsal neural tube (NT) before producing their own ECM (Perris, 1997; Perris and Perissinotto, 2000). The ECM provides critical signals that regulate NC cell migration, including permissive, non-permissive, and inhibitory components (Perris, 1997; Perris and Perissinotto, 2000).

Permissive signals, such as Fn1, laminins, and collagen I, promote strong cell adhesion and motility by interacting with integrin receptors on NC cells, facilitating cytoskeletal rearrangements and promoting migration (Duband, 2010; Szabó and Mayor, 2018). Non-permissive signals, such as certain collagen types and chondroitin sulfate proteoglycans, can provide weaker adhesion and modulate migration speed, often by restricting integrin activation (Szabó and Mayor, 2018). Inhibitory signals, largely proteoglycans like Vcan, can block NC migration by preventing integrin-mediated signaling, thus maintaining NC cells in a non-migratory state (Landolt et al., 1995; Henderson et al., 1997). These diverse ECM signals provide temporal and spatial regulation of NC migration during development. These ECM molecules, with their diverse permissive, non-permissive, and inhibitory properties, are crucial in guiding NC cell migration. In the context of zebrafish trunk NC cells, this complex interaction with the ECM becomes particularly evident. As these cells migrate along specific routes after delaminating from the neural tube, ECM proteins such as Fn1, laminin, and different type of collagens are essential for creating an environment that either facilitates or restricts cell movement (Banerjee et al., 2013).

Trunk NC cells in zebrafish migrate along specific routes after delaminating from the neural tube, transitioning from a sheet-like migration pattern to distinct cell streams as they reach the somite regions (Erickson, 1985). While somite-derived signals like Ephrin/Eph receptor and WNT signaling are known to regulate NC cell migration (Krull et al., 1997; Banerjee et al., 2011), the specific roles of ECM molecules *in vivo* are less understood. Fn1, laminin, and collagens are ECM proteins implicated in NC migration, with recent evidence suggesting that the enzyme lysyl hydroxylase 3 modulates trunk NC cell migration by post-translationally modifying non-fibrillar collagen, particularly Collagen 18A1 (Banerjee et al., 2013). Knockdown of collagen18a1 in zebrafish embryos leads to defects in NC cell migration, indicating that Collagen 18A1, possibly through interactions mediated by integrins or signaling domains, creates regions that are permissive or non-permissive for cell migration (Schneider and Granato, 2006; Myllylä et al., 2007). Additionally, MMP17b, expressed within NC cells, also plays a role in migration, potentially by cleaving ECM components or releasing guidance cues from the ECM (Leigh et al., 2013). These findings suggest that both ECM structure and its dynamic remodeling are critical for guiding the migration of trunk NC cells in zebrafish.

The ECM also mediates mechanical forces. Tissue stiffening occurs non-uniformly due to changes in cell density, cell adhesions, ECM composition, and matrix adhesion (Rozario and DeSimone, 2010).

**FIGURE 2:**
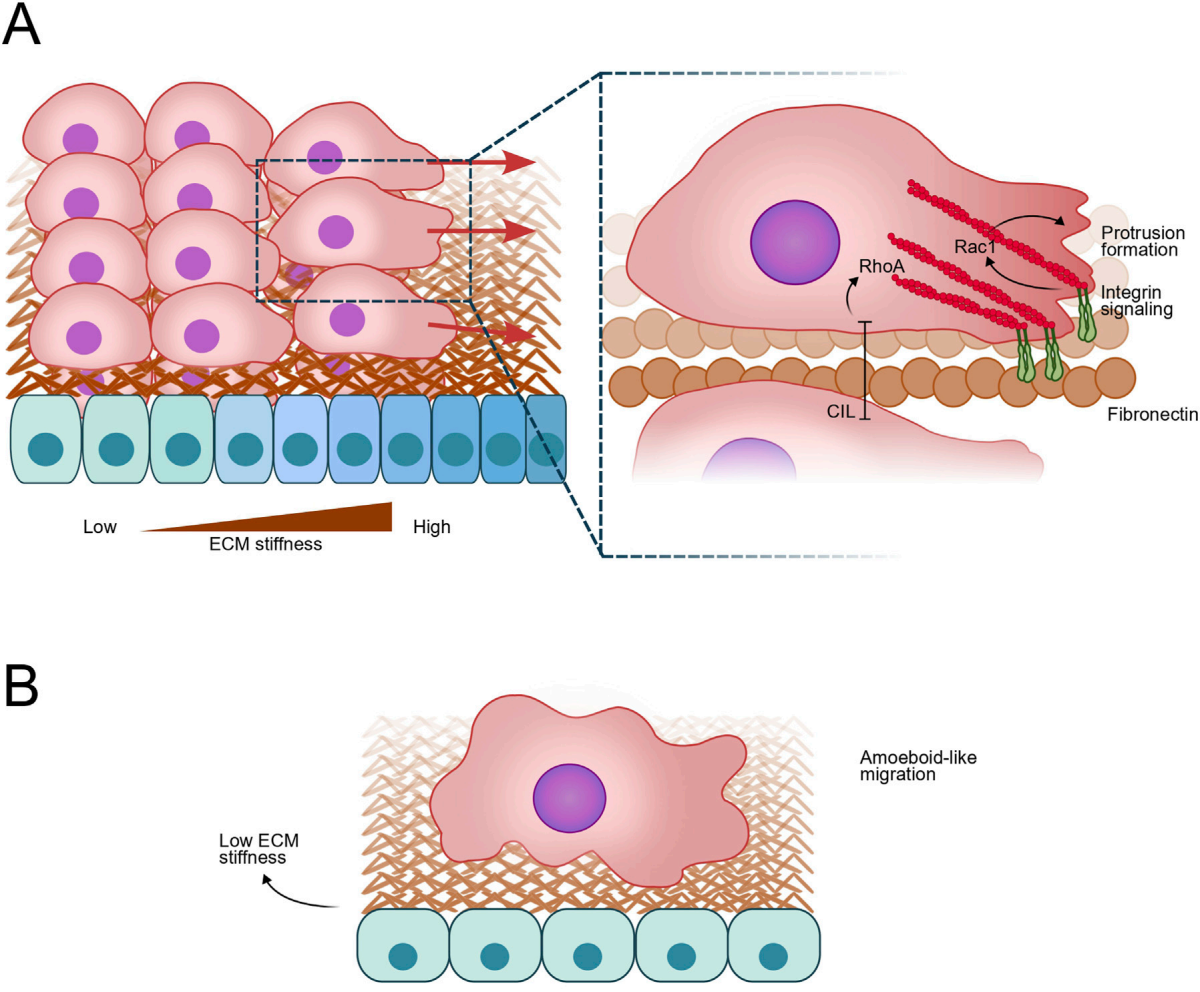
Neural Crest (NC) cell Migration in response to mechanical cues. **(A)** NC cells exhibiting collective migration in response to durotactic signals, characterized by a gradient of extracellular matrix (ECM) stiffness due to ECM composition, and cell density in the mesodermal tissue. This process involves integrin-mediated signaling and focal adhesion formation, leading to Rac1 activation and subsequently, actin polymerization and protrusion formation at the leading edge, while RhoA activation inhibit protrusion formation via Contact Inhibition of Locomotion (CIL). The mechanical properties of the ECM and mesodermal stiffness play crucial roles in guiding the directional migration of NC cells. **(B)** At low cell density, NC cells switch to an amoeboid-like migration, characterized by less dependence on focal adhesions and integrin signaling. Instead, this mode relies on the flexibility of the cell cortex and actomyosin contractility, allowing cells to move through the ECM with increased plasticity and adaptability. Amoeboid migration is facilitated by changes in cell shape and rapid, transient protrusions, enabling efficient navigation through variable ECM environments.

Recent studies highlight the significance of tissue mechanics during cell migration process, demonstrating that stiffening of the head mesoderm under the cranial NC cells triggers EMT and initiates collective migration (Kerosuo and Bronner-Fraser, 2012; Gilmour et al., 2017). Mechanosensing via integrin/vinculin/talin complexes allows NC to detect and respond to these changes in substrate stiffness (Gilmour et al., 2017). Moreover, convergent extension during gastrulation increases mesodermal cell density and stiffness, correlating with the onset of NC cell migration (Gilmour et al., 2017). These findings suggest a mechanical coordination between gastrulation and NC migration, bridging seemingly unrelated developmental processes through changes in tissue mechanics (Gilmour et al., 2017). Additionally, heterochronic tissue graft experiments demonstrate that environmental factors influence the timing of NC migration, implicating external cues in this migratory behavior (Theveneau et al., 2010). While Fn1, a major component of the NC ECM, remains unchanged during early developmental stages, *in vivo* measurements using atomic force microscopy reveal a gradual increase in mesodermal stiffness coinciding with NC migratory onset (Zhou et al., 2009; Koser et al., 2016). This stiffening of the mesoderm is strongly correlated with NC collective cell migration, suggesting that mechanical cues play a pivotal role in triggering and regulating this process *in vivo* (Koser et al., 2016) (Figure 2).

Mechanical cues play a crucial role in controlling the polarity of NC cells during migration. One of the primary mechanisms involves the asymmetric stiffening of cortical actomyosin networks, mediated by Rho GTPases and myosin II, which helps establish front-rear polarity in migrating cells (Vitorino and Meyer, 2008; Fischer et al., 2009). During collective migration, leader cells exhibit large, directed protrusions, which are essential for guiding the cluster. These cells are distinguished by their dynamic cytoskeleton and enhanced responsiveness to extracellular signals (Vitorino and Meyer, 2008). Additionally, the polarized remodeling of the ECM by these cells helps create paths for migration. For instance, cells degrade ECM components and lay down basement membrane components to facilitate movement (Smola et al., 1998; Schmidt et al., 2007). Cell polarity is also influenced by differential expression of ECM-binding proteins, such as integrins. Leader cells often express higher levels of integrins, which promote stronger attachment to the ECM and help define the direction of migration (Farooqui and Fenteany, 2005; Vitorino and Meyer, 2008) (Figure 2). *Ex vivo* experiments show that NC cells form large focal adhesions on stiff fibronectin-coated surfaces, contrasting with more amoeboid-like behavior observed in softer *in vivo* environments (∼120 Pa stiffness in *Xenopus* embryos) (Barriga et al., 2018) (Figure 2). This competition between focal adhesions and the cell cortex for actomyosin machinery recruitment influences cell movement adaptability. Surprisingly, similarities exist between single-cell amoeboid “swimmer” migration and contractility-driven collective migration models, suggesting NC cells may exhibit more amoeboid-like behavior than previously thought, especially under softer *in vivo* conditions (Carragher et al., 2006; Liu et al., 2015) (Figure 2). *In vivo*, NC migration responds to mesodermal stiffening through integrin-mediated complexes involving vinculin and talin, influencing protrusion dynamics and collective migration model (Carmona-Fontaine et al., 2008; Barriga et al., 2018). Various mechanisms such as rear actomyosin contraction, confinement, CIL, protrusions, and mechanical cues from the mesoderm collectively orchestrate efficient migration (Theveneau et al., 2010; Carmona-Fontaine et al., 2011; Scarpa et al., 2015; Roycroft and Mayor, 2016).

CIL further ensures proper cell polarity. Upon contact, cells collapse their protrusions at the site of contact, repolarize, and extend new protrusions away from the contact site. This process involves various cell-cell adhesion molecules, including cadherins and ephrins, and leads to the activation of small GTPases such as RhoA, Rac1, and Cdc42. RhoA is activated at contact sites, while Rac1 and Cdc42 are inhibited at these sites but activated elsewhere, promoting new protrusion formation and cell polarization (Astin et al., 2010; Mayor and Carmona-Fontaine, 2010).

Thus, mechanical cues, through actomyosin network dynamics, ECM remodeling, integrin expression, and CIL, critically control the polarity of NC cells, ensuring efficient and directed migration.

### Congenital diseases produced by defects in neural crest migration and differentiation

The regulation of NC cell migration, proliferation, and differentiation is tightly controlled by multiple signaling pathways, and disruptions in these pathways can result in neurocristopathies—developmental disorders arising from defects in NC cells. As we mention in the previous section, signaling pathways such as RA, BMP, TGF-β, NOTCH, WNT, and Sonic Hedgehog (SHH) play critical roles in NC development by establishing cell polarity, influencing cytoskeletal dynamics, and directing cell migration, essential for proper tissue formation. Disruptions in these pathways can cause neurocristopathies by altering the polarity and migration of NC cells (Sauka-Spengler and Bronner-Fraser, 2008; Scholl and Kirby, 2009; Mayor and Theveneau, 2014; Shih et al., 2017).

The BMP/TGF-β signaling pathway plays a critical role in regulating bone differentiation, and mutations in genes associated with this pathway are linked to bone and cartilage developmental disorders. For example, abnormal BMP signaling in NC cells can result in the formation of ectopic cartilage in cranial sutures, leading to their premature fusion (Ueharu et al., 2023).

The effects of NOTCH signaling and its ligand JAG1 are associated with congenital disorders like Alagille syndrome, which is characterized by cardiac, biliary, and skeletal abnormalities (Penton et al., 2012). In cranial NC cells, depletion of Jag1 leads to a smaller maxilla, abnormal vascular branching, reduced cell proliferation and decreased extracellular matrix production, highlighting the essential role of Jag1 in craniofacial development, vascular formation, and tissue growth (Humphreys et al., 2012). Furthermore, the JAG1 through NOTCH1 non-canonical pathway activates osteoblast-specific gene expression in cranial NC cells, promoting osteoblast differentiation and facilitating bone mineralization (Kamalakar et al., 2021).

NC cells migration to the anterior part of the sclerotome, allow the migration and location of these cells at different levels of the gut (O’Leary and Wilkinson, 1999). Then NC cells differentiate into Auerbach’s and Meissner’s ganglia to form the enteric nervous system. The absence of these enteric ganglion cells produces the congenital disease known as Hirschsprung’s or congenital aganglionosis (Howard and Garrett, 1970; Furness, 2012).

Another example is Bardet–Biedl syndrome (BBS), where patients exhibit craniofacial defects and Hirschsprung’s disease, among other symptoms. In zebrafish, it has been demonstrated that the genes whose mutations cause this syndrome—BBS4, BBS6, and BBS8—are required for proper NC migration, which could be explained by an aberrant SHH signaling (Tobin et al., 2008).

Cleft lip with or without cleft palate (CLP) malformation also results from aberrant NC cell migration. In this case, the combination of loss of function of CDH1/E-cadherin and a proinflammatory environment, which leads to hypermethylation of CDH1/E-cadherin, causes defects in NC cells migration (Alvizi et al., 2023).

Other conditions derived from aberration in NC cell migration and differentiation is the Retinoic Acid Syndrome (RAS), which show developmental abnormalities of the mandible (micrognathia) and palate (cleft palate), facial nerve palsy, absent or deficient thymus and parathyroid glands (Sulik et al., 1988; Johnston and Bronsky, 1995; Morriss-Kay and Wardt, 1999). Additionally, it was found that the absence of RA signaling pathway results in the loss of FGF8 and SHH signaling causing cell apoptosis and inhibition of cell proliferation. Cell apoptosis mediated by p53, as well as FGF8 and SHH signaling, leads to a deficiency in cranial NC cells resulting in cranioskeletal hypoplasia observed in patients with Treacher Collins Syndrome (Trainor, 2010). These suggest that exist a critical period during the NC cells migration and differentiation that dependents on RA signaling for proper embryo development (Johnston and Bronsky, 1995; Laue et al., 2011; Rhinn and Dollé, 2012).

In addition, defects in the NC cells migration can lead to alterations in cardiac development, particularly affecting the formation of the OFT and aortic arch (AA). These defects can cause congenital heart diseases such as persistent truncus arteriosus, resulting from incomplete OFT formation, and tetralogy of Fallot, resulting from OFT misrotation (Maeda et al., 2006). Developmental anomalies of the AA system arise from regression of parts of the pharyngeal arch arteries. For example, interrupted AA type B results from regression of the left pharyngeal arch artery, while aberrant right subclavian artery results from the regression of the right fourth pharyngeal arch (Yamagishi, 2021).

Furthermore, mutations in different signaling pathways involved in cardiac NC cell migration can lead to defects in pharyngeal arch arteries. For instance, mutations in TGF-β and BMP signaling pathways, which interact with Smad proteins can cause such defects (Molin et al., 2004; Nie et al., 2008). Additionally, mutations in the MAML gene under the Pax3 promoter block NOTCH signaling, which inhibits the differentiation of smooth muscle in the pharyngeal arch arteries and results in the failure of cardiac NC cells migration and differentiation into smooth muscle (Huang et al., 2008; Varadkar et al., 2008; Manderfield et al., 2012).

Krox20, a transcription factor plays a crucial role in hindbrain patterning and morphogenesis, by binding to specific DNA sequences in the 5′ flanking regions of genes such as Hox2, Hoxb2, Hoxb3, and Eph4 (Lemaire et al., 1988; Nardelli et al., 1991). This binding directly controls the expression of these genes. Targeted mutations of Krox20 in mouse embryos result in perinatal death, abnormal fusion of trigeminal ganglia with facial and vestibular ganglia, and hyperplastic aortic valve formation, leading to bicuspid aortic valves (Schneider-Maunoury et al., 1993; Swiatek and Gridley, 1993; Odelin et al., 2018). Additionally, multiple alterations or deletions of Hox genes such as Hoxa3 and Hoxad3, cause abnormalities in cartilage and laryngeal muscles, leading to DiGeorge syndrome, as well as hypoplasia and the absence of the thymus and parathyroid glands (Chisaka and Capecchi, 1991; Condie and Capecchi, 1994; Manley and Capecchi, 1995).

The cellular and molecular mechanisms governing NC cell behavior play a crucial role in the formation of the peripheral nervous system, as well as the connective, bone, cartilage, and muscle tissues of the embryo. Disruptions or aberrations in these mechanisms can lead to a range of congenital diseases, as discussed in this section. However, the precise interactions between signals required for proper embryonic development are still not fully understood.

## Conclusion and perspectives

The migration and differentiation of NC cells are highly regulated processes involving a complex interplay of molecular and mechanical signals. The intricate balance between molecular signals and mechanical forces that regulates NC cell behavior underscores the complexity and precision required for their proper migration (Sauka-Spengler and Bronner-Fraser, 2008). These processes are not only essential for proper organogenesis but also provide a fascinating model for understanding how migrating cells interpret and respond to a multitude of signals. Establishing and maintaining correct cell polarity is essential for directional migration and the response to environmental cues. As mentioned in this review, key regulators of this process include small GTPases such as Rac1, Cdc42, and RhoA (Rodriguez et al., 2003; Lawson and Ridley, 2018) and the PCP complex, which arrange cell protrusions and contractility, guaranteeing effective migration (Theveneau and Mayor, 2010). The interplay between these GTPases, along with actin filaments and microtubules, governs the structural and motility aspects of NC cells. Heterotrimeric G proteins and Ric-8A also play essential roles in modulating cell adhesion, chemotaxis, and polarity during NC cell migration. Ric-8A regulates the localization of active Rac1 and RhoA, connecting G-protein signaling to cytoskeletal dynamics and cell shape (Fuentealba et al., 2013; Toro-Tapia et al., 2018; Leal et al., 2018). Another factor influencing NC cell migration is the ECM composition and stiffness (Bonnans et al., 2014). Stiffening of the mesodermal environment triggers EMT and collective migration. Mechanical cues, such as tissue stiffness, guide NC cells through integrin-mediated signaling complexes, enabling their response to environmental changes (Carmona-Fontaine et al., 2008; Barriga et al., 2018). Defects in NC cell polarity during migration and differentiation result in several congenital disorders, such as neurocristopathies and cardiovascular anomalies, highlighting the importance of understanding these mechanisms (Maeda et al., 2006; Alexander et al., 2009; Barber and Rastegar, 2010). In summary, the migration of NC cells is a multifaceted process regulated by a network of signaling pathways, cytoskeletal dynamics, and ECM interactions. Further research is needed to elucidate the specific molecular interactions between the signaling pathways involved in NC cell migration and polarity and ECM homeostasis. Additionally, the EMT is a process that is also crucial in cancer metastasis. EMT enables cancer cells to acquire migratory and invasive properties like NC cells (Gundamaraju et al., 2022), highlighting parallels in the mechanisms of cell migration across different contexts.

Understanding these processes will provide insights into the developmental origins of neurocristopathies and contribute to the development of new therapeutic strategies for related congenital diseases and other pathologies resulted by similar defect like cancer.
